# Antagonistic pleiotropy as a widespread mechanism for the maintenance of polymorphic disease alleles

**DOI:** 10.1186/1471-2350-12-160

**Published:** 2011-12-12

**Authors:** Ashley JR Carter, Andrew Q Nguyen

**Affiliations:** 1 Biology Department, California State University Long Beach, 1250 Bellflower Blvd, Long Beach, CA 90840, USA

## Abstract

**Background:**

Many serious diseases have a genetic basis which, from an evolutionary point of view, should have been selected against, resulting in very low frequencies. The remarkable sustained prevalence of a number of disease-associated alleles is therefore surprising. We believe that antagonistic pleiotropy, when multiple effects of a gene have opposing effects on fitness (e.g., sickle cell disease), may be more widespread than typically considered. We hypothesize that, rather than being an exception to the rule of genetic disorders, antagonistic pleiotropy may be common.

**Methods:**

We surveyed the medical literature in order to determine whether sufficient evidence exists to reassess the nature of antagonistic pleiotropy; from being considered an unusual scenario to one that is anticipated. We also used a simple population genetic model to examine the feasibility of antagonistic pleiotropy to act as a mechanism to maintain polymorphism for serious genetic disorders even if the benefits are subtle.

**Results:**

We identified a number of examples of antagonistic pleiotropy where the deleterious effect, the beneficial effect, and the exact molecular cause have been demonstrated. We also identified putative cases in which there is circumstantial evidence or a strong reason to expect antagonistic pleiotropy in a genetic disorder. The population genetic model demonstrates that alleles with severe deleterious health effects can be maintained at medically relevant frequencies with only minor beneficial pleiotropic effects.

**Conclusion:**

We believe that our identification of several cases of antagonistic pleiotropy, despite the lack of research on this question and the varied natures of the types of these disorders, speaks to both the underappreciated nature of this phenomenon and its potentially fundamental importance. If antagonistic pleiotropy is as common as our research suggests, this may explain why so many serious diseases, even apparently environmentally caused ones, have a genetic component. Furthermore, acceptance of a genome full of antagonistically pleiotropic genetic interactions poses important implications for clinical treatment and disease prevention research, especially genetically based therapies.

## Background

The human genome is the product of an evolutionary history that combines mutational processes and natural selection; since this process appears to be a very effective mechanism for optimizing many traits (i.e., adaptation) it is reasonable to question why so many genetic disorders (i.e., non-optimal alleles) exist. Genetic disorders are typically defined as «mutant» alleles that confer deleterious effects on their possessors (usually, but not always, homozygotes for the mutant allele) relative to those possessing wildtype or normal alleles. Although genetic drift and mutation selection balance can explain the presence of segregating allelic variation for deleterious traits, many genetic disorders are present at frequencies higher than we would expect from a historical process by which mutations arise and are then eliminated by selection over time. A potential resolution to the discrepancy between our expectations of very low rates of genetic disorders and our observations of remarkably common genetic disorders involves considering the other effects of these mutant alleles aside from just the deleterious ones.

In contrast to long-held notions whereby single genes encoded single functions (e.g., «the» eye color gene), most genes are now recognized to have multiple qualitatively distinct functions, a phenomenon termed pleiotropy [1]. A simple thought experiment where we consider that the human genome has approximately 23,000 genes (with multiple genes involved in the formation of each aspect of our phenotype) encoding a phenotype with more than 23,000 aspects demonstrates that many genes must have multiple functions. This being the case, allelic variants that arise due to mutation may change the performances of that gene's functions differently such that sometimes these changes result in opposing effects on fitness - i.e., some mutations can improve the performance of one aspect of a gene's function while simultaneously making another worse. Such a phenomenon is termed antagonistic pleiotropy, the pleiotropic functions of the same gene act antagonistically in terms of the fitness of the possessor. To date, consideration of the implications of such antagonistically pleiotropic effects has largely been the purview of studies of senescence.

Senescence is associated with distinct changes in the physiology of individuals as they age [2]. Natural selection acts on differences in relative fitness (which is comprised of both mortality and reproduction) in individuals within populations; the lack of natural selection to completely eliminate many deleterious senescence-associated traits explains their sustained prevalence in populations of humans. The most widely held evolutionary explanation for the continued presence of senescence incorporates the aforementioned antagonistic pleiotropy; an allele that causes deleterious traits can actually be selected to increase in frequency over time if the deleterious effects on the fitness of the possessor are outweighed by other advantageous effects also due to the allele [3].

Natural selection acts the strongest on traits manifested during an organism's peak reproductive value, an age that corresponds to the beginning of reproduction in most species (i.e., sexually mature, but young), and benefits to an organism during this time increase overall fitness more than those manifested at other ages [1]. In contrast, natural selection is the weakest on traits manifested after the majority of an organism's reproduction is complete (i.e., middle-aged or elderly), and detriments to an organism during this time reduce overall fitness less than those manifested at earlier ages. Pleiotropically antagonistic alleles that provide a net increase in fitness therefore tend to be those with benefits expressed at a young age, favoring either fertility or survival, while the detrimental phenotypes are not expressed or do not display high penetrance until post-reproduction. A long history of selection for, and fixation of, such antagonistically pleiotropic alleles is thought to account for biochemical pathways and physiological processes that work well in youth, but less well in older age - i.e., senescence [1,3].

Previous studies have discussed the influence of senescence on different human diseases such as atherosclerosis [4], sarcopenia [5], osteoporosis [6], immunodeficiency disorders [7] and neurodegenerative disorders such as Alzheimer's([8] (see also a review in [9]). In fact, a number of alleles associated with a range of other diseases may have possessed similar antagonistically pleiotropic effects and led to specific aspects of our senescence. For example, it has been argued that positive selection and sexual conflict may account for increased carcinoma risk [10]. They reason that higher selective pressures due to sexual and generational conflict resulted in positive selection, as suggested by relatively high rates of nucleotide and amino acid sequence evolution in genes associated with such conflicts, and that these changes had maladaptive pleiotropic side-effects that increase the rate of carcinoma [10]. We suggest that in addition to antagonistic pleiotropy contributing to the fixation of carcinoma causing mutations it may contribute similarly to current polymorphisms of other alleles associated with cancer and other non-cancerous conditions.

Previous consideration of this topic has tended to focus on selection that resulted in the fixation of alleles that cause their deleterious effects in older age, changing the default (i.e., wildtype) condition in the population. In actuality, natural selection is often a slow and gradual process and many alleles that differ in their effect on fitness can be present in populations for prolonged periods, especially if the overall fitness differences are small. For example, an allele that confers a noticeable disadvantage at one stage of life may also favor the individual at another stage of life and if the net effect is small then selection to change the allele's frequency will be weak and the frequency will be changed slowly by selection. Natural selection can also act to actively maintain alleles with individually deleterious effects in populations if they act synergistically to increase fitness when present together (e.g., via heterosis or epistatic interactions).

A clear example of a medically relevant antagonistically pleiotropic polymorphic allele was recently described [11]. They conducted a cohort study of 99 individuals in Ecuador with Laron Syndrome, a rare form of Dwarfism. Individuals with Laron Syndrome may possess one of three different genotypes relating to their Growth Hormone Receptor gene (GHR); most individuals carry an A to G splice site mutation in position 180 in exon 6 while some carry a nonsense mutation designated as R43X and others are heterozygous for these two mutations. That study followed Laron Syndrome patients and their non-dwarf kin from 1988 to 1998 and recorded medical information. The study found that patients with Laron Dwarfism experienced virtually no incidences of cancer whereas their non-Dwarf kin experienced a 17% cancer mortality rate. Laron dwarfs also seemed to be protected from diabetes; no incidences were reported among Laron patients whereas their kin suffered a 5% mortality rate. The presence of the various mutant GHR alleles in this population may be due to drift (the population is quite small and relatively isolated), but may also be due to selection acting to maintain this allele in the population due to the benefits provided. While the allele described in this case is rare and represents a minor overall effect on health and fitness of the wider population, if this general phenomenon of antagonistic pleiotropy is more common it may account for disorders that have more widespread impact. The recognition that alleles identified as being deleterious may also provide benefits is one that we believe has been underappreciated in the medical literature.

The purpose of this investigation is to determine whether evidence of balancing selection due to antagonistic pleiotropy, heterosis or epistatic interactions is seen in alleles associated with increased risk of certain human diseases. As the phenomena of heterosis and beneficial epistatic interactions are likely to arise from the multi-functional nature of genes, we use the term antagonistic pleiotropy as a catch-all term to refer to alleles that act in any of these manners. If the net relative fitness conferred by pleiotropically antagonistic alleles with mixed effects is higher, or even just comparable, to wildtype individuals then such alleles would likely continue to persist in the population. If mutations that create such pleiotropic alleles are not extremely rare, widespread antagonistic pleiotropy may explain the maintenance and high prevalence of many disease-associated alleles. It becomes of great importance therefore to determine whether antagonistically pleiotropic alleles are likely to be rare or may be more common than previously realized.

## Methods

In the body of this manuscript, evidence of antagonistic pleiotropy in several major diseases is presented on a disease by disease basis. The pleiotropic alleles, their benefits and their pathophysiological costs are all briefly outlined. Our review of the research literature supports the hypothesis that at least some of the genetic factors that increase disease severity or risk may also confer certain reproductive or survival benefits, and therefore provides support for the importance of antagonistic pleiotropy as a causal mechanism for the persistence of disease causing alleles. We would also like to stress that, due to the lack of attention paid to the potential benefits of alleles associated with different pathologies, we believe that our collection of examples may only touch the surface of the phenomenon and is a conservative estimate of its prevalence. After describing a number of conditions in detail (summarized in Tables 1 and 2) we discuss possible implications of widespread antagonistic pleiotropy for current and proposed medical treatments, clinical trials and research.

**Table 1:** Antagonistically pleiotropic disease alleles.

Deleterious effect (disease)	Gene: variation	Benefit
Increased risk of cancers: ovarian (females), prostate (males).	AR: CAG trinucleotide repeat lengths.	Females: increased fertility via reduced degree of pre-mature ovarian failure, reduced risk of breast cancer. Males: increased number of viable sperm, reduced risk of Kennedy's Disease.

Huntington's disease.	HTT: CAG trinucleotide repeat lengths.	Increased fertility, decreased risk of certain cancers.

Sickle cell disease.	Hbb: various point mutations.	Protection against malaria in the heterozygous state.

Beta-thalassemia.	HB: various point mutations	Protection against malaria in the heterozygous state.

Glucose-6-phosphate dehydrogenase (G6PD) deficiency.	G6PD: various missense mutations.	Protection against malaria.

Cystic fibrosis (CF)	CFTR: missense mutation.	Increased fertility.

Increased risk of osteoporosis in old age.	ALOX15: A:G intronic polymorphism.	Reduced risk of osetoporosis in youth.

These diseases are described in more detail in the text, the presence of an advantage in addition to the deleterious effects of the disease is well-supported by detailed genetic or epidemiological information.

**Table 2:** Putative antagonistically pleiotropic disease alleles.

Disease	Gene; Variation	Putative Benefit
Triosephosphate Isomerase Deficiency	TPI Gene: point mutations.	Reduction in oxidative stress.

Tay Sachs	HEXA: missense mutations.	Protection against tuberculosis.

Hemachromatosis	HFE Gene: many point mutation.	Better iron absorption, increased resistance to typhoid fever and tuberculosis

Phenylketonuria	PKU: missense mutation.	Lower probability of miscarriage

Leukemia	PTNP11: various expression level mutations.	Lower probability of hepatocellular carcinoma

Schizophrenia	Chromosomal regions 6p22-p24 and 11q21-22.	Higher relative fertility (in unaffected relatives).

Cancer	TNFRSF11B Gene; Various Polymorphisms	Increased Bone Density in Females

These diseases have been proposed to have antagonistic pleiotropic effects in their etiology, but the evidence is suggestive and preliminary.

## Results

### Huntington's disease and Increased Fecundity

Huntington's disease (HD) is a rare, autosomal dominant neurodegenerative disorder in which symptoms typically manifest at an age that is post-reproductive, in the range of 30-45 years of age [12]. Affected individuals often experience chorea, uncontrolled and involuntary movements, in addition to a much shorter life-expectancy. Onset of Huntington's disease is attributed to the presence of a high number of CAG trinucleotide repeat lengths within the Huntingtin (HTT) gene [12,13]; in particular, when the number of CAG repeats exceeds 37, onset of Huntington's disease symptoms was much more likely to occur [14].

Huntington's patients show a notable increase in fecundity when compared to the unaffected individuals however, and may also experience lower rates of cancer. In a study of the relative fitness of affected and unaffected individuals in Canada via a comparison of the fitness values of 157 Huntington's patients with 170 related wild-type individuals, the mean number of offspring for the patients was 39% higher than their unaffected siblings and 18% higher than a set of unrelated control individuals matched for age and sex [15]. That investigation went on to predict that if such fitness values as he observed were genuine and were to persist, the disease-associate polymorphisms may double in frequency in as short as 150 years (roughly six generations)[15].

An additional benefit that may be conferred by these antagonistically pleiotropic polymorphisms is a reduced risk of cancer [16]. Sorenson et al [17] reported that a higher number of CAG repeats within the HTT allele is associated with increased p53 activity. P53 is a tumor suppressor protein that has a role in inducing apoptosis and although apoptosis of neuronal cells may be the major cause of the neuro-degeneration seen in Huntington's patients, a higher level of p53 may also protect from tumor growth and cancer development in general. The increased CAG repeat length of HD individuals may therefore be an explanation for the reduced incidence of cancer seen in a large registry of Huntington's patients [17].

Variation in the CAG repeat length within the HTT gene therefore seems to exhibit a form of antagonistic pleiotropy that perfectly illustrates that proposed by [3]. Presence of more than 37 CAG trinucleotide repeats in the HTT gene is associated with an increased risk for Huntington's disease, but is also associated with a substantial rise in fertility and perhaps reduced cancer risk.

### Cystic fibrosis and fertility

Cystic fibrosis (CF) is an autosomal recessive trait that is often lethal, though life expectancy has increased over time from early infancy to late-30s due to better medication, therapies and more access to medical care [18]. The mutation that causes CF is a codon deletion in the CFTR gene that results in the loss of phenylalanine from the protein product [19]. Loss of phenylalanine results in a non-functional trans-membrane chloride channel, a channel responsible for the movement of bodily fluids throughout many different organs and organ systems in the body. The presence of this defective channel in the body leads to diverse symptoms involving many organ systems and tissues, mainly due to the buildup of fluids throughout the body. The backup of fluid contributes to other serious ailments including increased susceptibility to infections and various breathing problems [20].

Alleles associated with cystic fibrosis may confer certain benefits via overdominance (i.e., heterozygotes have higher fitness) or epistatic effects however. Data shows that individuals in families with members identified as suffering from CF have more offspring than individuals from control families [21]. Even though the offspring from CF family members experienced higher mortality rates than the offspring of the non-CF family individuals, CF family individuals still out-reproduced non-CF family individuals by roughly 22% (p < 0.001)[21].

Additionally, using a mouse model, an investigation conducted by Gabriel and colleagues [22] showed that heterozygotes for non-functioning CFTR alleles were resistant to cholera toxin which suggests that increased resistance to cholera may also explain the persistence of CF. These mouse results were similar to results obtained from human tissue cultures [23], however that study did not explicitly demonstrate a fitness benefit to CF heterozygotes in humans. Finally, an advantage arising from decreased levels of sialic acid on cell membrane surfaces in CF heterozygotes was proposed [24]. This decrease could potentially provide increased resistance to influenza and other myxo- and paramyxoviruses, although as above, cell tissue culture data instead of direct comparisons of fitness differences between CF heterozygotes and other individuals were used to support this hypothesis [24].

The allele with the deleted CFTR codon therefore seems to exhibit antagonistic pleiotropy. The deletion leads to serious health issues in homozygotes, but individuals likely to be carrying the allele (I.E., families of CF sufferers) appear to have more offspring and may experience increased resistance to cholera and other infectious diseases.

### Sickle Cell Anemia and malaria resistance

Sickle cell disease is an autosomal recessive hemoglobinopathy commonly found in individuals endemic to tropical and Mediterranean regions, especially Africa [25]. Sickle cell disease is attributed to a single missense point mutation in the Hemoglobin (Hb) gene, termed the Hb-S allele. The mutation leads to the replacement of glutamine by valine at position 6 and to the development of the characteristic abnormal sickle shaped erythrocytes that are associated with sickle cell anemia. The sickle shape compromises the elasticity of the red blood cell and causes these cells to obstruct blood flow through narrow vessels, resulting in ischemia and other serious problems. Although life-expectancy of individuals with the homozygous Hb-S genotype has increased in recent years in developed nations (median lifespan of 42-48 in the US) [26], for most of human history (and even now in undeveloped nations) homozygous individuals had very short expected life expectancies (I.E., high juvenile mortality). In contrast, individuals who are heterozygous for the Hb-S allele do not present with full symptoms and suffer minimal hemoglobinopathies.

The Hb-S allele provides an advantage in individuals heterozygous for the Hb-S allele via increased resistance to malaria, most notably during the ages of 2 to 16 months [27]. The presence of such a benefit early in life is thought to account for the relatively higher frequency of the sickle cell allele in geographic regions that experience high rates of malaria. Malaria is estimated to infect approximately 400 million individuals and kill over one million each year [28]. Although the high rate of malaria is a relatively recent phenomenon, it has been a major health factor for a large proportion of humanity for over 500 generations and therefore has had sufficient time to change the frequencies of alleles associated with resistance [28].

The Hb-S allele is perhaps the best known and most widely used example of antagonistic pleiotropy in discussions of the topic in human disease due to the clear deleterious effect in homozygotes combined with a clear advantage in heterozygotes. In contrast to the previous examples the advantages and disadvantages of this allele are not experienced by the same individuals, but the overall selective forces acting on the allele remain very similar. Over mankind's evolutionary history (and in the present) selection both favors and acts against the Hb-S allele simultaneously, resulting in an equilibrium at a frequency much higher than that expected for a purely deleterious allele.

### Beta-Thalassemia and malaria resistance

Another example of antagonistic pleiotropy in a hemoglobinopathy is the blood disorder Beta-Thalassemia which is caused by a mutation in the beta chains of hemoglobin [29]. Beta-Thalassemia is an autosomal, incompletely recessive, mutation in which individuals that are homozygous for the mutation often present with full disease symptoms (known as Beta-Thalassemia Major). Beta-Thalassemia Major symptoms include anemia, some heart/vascular problems in adults, and infants and newborns that are often underweight and smaller in size than average children (experiencing the condition termed «failure to thrive») [29]. Although heterozygotes for the mutation often present with mild symptoms of this disorders, they seem to suffer no difference in life expectancy relative to the general population [30].

A study reported that the possession of the Beta-Thalassemia mutation is associated with increased resistance to malaria and that the basis for malarial protection may be due to early childhood exposure to *P. vivax *resulting in resistance to *Plasmodium falciparum *[31]. The protection from malaria increases the relative fitness of these individuals greatly and is thought to account for the relatively higher frequency of the Beta-Thalassemia mutation in geographic regions that experience high rates of malaria.

The Beta-Thalassemia anemia variant of hemoglobin B mirrors the case of sickle cell anemia described above due to the clear advantage for heterozygotes and the clear deleterious effects in homozygotes. Interestingly, while the sickle cell and malaria example is more widely known and taught in biology courses, the argument for the role of malaria in the maintenance of the Beta-Thalassemia mutation via an overdominance based antagonistic pleiotropy mechanism predates it [31,32].

### Glucose-6-Phosphate Dehydrogenase Deficiency and malaria resistance

Glucose-6-Phosphate Dehydrogenase (G6PD) deficiency is a genetic disorder that often results in hemolytic anemia because G6PD is essential for the prevention of hemolysis due to reactive oxygen species accumulating in red blood cells during regular cellular metabolism and from exposure to certain environmental stimuli. A number of observed mutations to the G6PD gene cause a truncated dehydrogenase enzyme that is severely compromised in function and with an estimated 400 million affected individuals this disorder is one of the most common among enzyme deficiencies [33]. Homozygous (females) and hemizygous (males) individuals manifest the effects of the recessive mutation which often results in fetal mortality and, later in life, causes hemolytic anemia if red blood cell production cannot be increased enough to offset the rate of destruction from hemolysis. Current studies show that the life expectancy of affected individuals can be normal with adequate and frequent treatment [33,34], although this treatment is not available in much of the world and was not available throughout most of medical history.

As is the case for the hemoglobinopathies described above, G6PD deficiency is associated with protection from malaria. Various alleles that cause G6PD deficiency have been associated with improved resistance to malaria, especially to the most life-threatening cerebral malaria, in hemizygous males and heterozygous females [35,36].

Although they cause hemolytic anemia, the resistance to such a widespread and serious disease conferred by G6PD mutations may cause the net fitness effect of these alleles to be comparable to wildtype fully functional G6PD alleles. The high prevalence and serious health consequences of malaria infection may explain the extremely high number of cases of G6PD estimated to exist.

### ALOX15 gene and Bone Mineral Density

Osteoporosis is a condition in which the bones weaken, making sufferers more prone to fractures in accidents and falls, and poses a serious global health risk. For example, in the US in 2005 there were an estimated 300,000 hip and 550,000 vertebral fractures [37] and in a Canadian cohort study these fractures were associated with 419% and 253% increases in mortality over the next year respectively [38]. Clinical definition of osteoporosis typically includes low bone mineral density (BMD) measurements. In a study of SNP frequencies the presence of a guanine instead of the more common adenine within intron 12 of the 12/15-lipoxygenase (ALOX15) gene was associated with a reduced risk of low BMD at the femoral neck in pre-menopausal women (OR = 0.442, p = 0.007) but an increased risk in postmenopausal women (OR = 1.727, p = 0.042) [39]. The guanine allele therefore either reduces or increases osteoporosis risk depending on which stage of life one is considering; if we consider the actions of ALOX15 during different life stages to be different functions then this allele is a clear case of an antagonistically pleiotropy.

### Cancers and reproductive traits

Cancer is a primary cause of genetically caused mortality worldwide, with estimates of up to 569,490 deaths in 2010 in the US alone [40]. A number of allelic variants for certain genes have been discovered in which the genotypes of individuals seem to correlate with risk of developing cancer [41]. One of these genes is the androgen receptor (AR) gene. The AR gene contains a repeated CAG trinucleotide sequence and the CAG repeat length influences gene expression, which determines the density of androgen receptors in human epithelial tissue, mainly of the reproductive system.

In females, a shorter CAG repeat length in the AR gene is associated with ovarian cancer. Shorter repeats of the CAG segment have been associated with an increased expression of the AR gene in ovarian tissue [42,43] and another study presented a Western Blot analysis of tissue cultures as evidence that increased expression of AR gene is associated with ovarian tumor formation [44]. Consistent with this process, women who inherited a shorter repeat of the CAG segment of the androgen receptor allele presented with an earlier age of incidence of ovarian cancer, roughly about 7.2 years earlier compared to a control group [42].

In males, a shorter CAG repeat length in the AR gene is associated with prostate cancer. As in ovarian tissue, a shorter CAG repeat length is associated with increased expression of the AR gene in prostate tissue [45]. Higher density of AR in prostate tissue seems to amplify androgen activity, which is expected to increase cell proliferation and prostate tissue growth rates [46]. Increased cell proliferation, via hormonal stimulation for example, is associated with an increase in frequency and malignancy of prostate cancer [43,47]. Consistent with this process, Ingles and colleagues reported that males with a shorter repeat length experienced a two-fold increase in prostate cancer risk [46]. Similarly, another investigation reported an association between shorter CAG repeats and prostate cancer in 587 prostate cancer patients and 588 control individuals and that the association was driven by the most severe cases [48].

In contrast, shorter repeat CAG lengths within the AR gene may provide benefits to females and males.

In females, shorter repeats may increase reproductive ability and reduced breast cancer risk. Shorter CAG repeat lengths within the AR gene are associated with a potential increase in reproductive fitness via the link between AR gene expression and follicular development during the uterine cycle. Serum androgen activity is mediated by the androgen receptor and the effects appear additive, implying that a high density of the receptor (such as that caused by increased expression due to shorter repeat lengths) would amplify androgen activity. Androgen activity is associated with follicular growth and maturation and increases the viability of oocytes, which increases fertility [49]. Consistent with this process is evidence provided by Chaterjee and colleagues in which shorter CAG repeat lengths were associated with a reduced degree of pre-mature ovarian failure [50]. Shorter repeat lengths may also provide an advantage via reduced cancer risk; in a study of women with the BRCA1 allele, shorter CAG repeat lengths within the AR gene were associated with reduced risk of breast cancer [51].

In males, shorter CAG repeat lengths within the AR gene are associated with a potential increase in reproductive ability, reduced risk of Kennedy's disease and perhaps greater sexual attractiveness. There is evidence of an inverse relation between CAG repeat lengths in the AR gene and the level of reproductive fitness; on average, males with a shorter CAG repeat length presented with fewer non-viable, defective sperm [52]. The level of severity and the associated amount of defective sperm were directly correlated to longer repeat lengths and individuals with shorter repeats experienced reduced rates of infertility. There is also evidence that a shorter repeat length is associated with increased counts of viable sperm in adolescent males [53]. Shorter repeat lengths may provide an advantage via reduced risk of Kennedy's disease, a neuromuscular disease similar to ALS that only affects males. In a study of 35 unrelated men with Kennedy's disease and 75 controls [54] all the affected males had longer CAG repeat lengths. It appears that a decreased repeat length is associated with higher expressions of a range of male- specific traits that may have had (or indeed, still have) roles in mate acquisition and attraction.

Variation in the CAG repeat length within the AR gene therefore seems to exhibit antagonistic pleiotropy in both females and males. In females, shorter CAG repeat lengths correlate with increased risk of ovarian cancer, the second leading cause of gynecological cancer deaths [55], yet shorter repeats are also associated with increased reproductive fitness via increased viability of oocytes and reduced pre-mature ovarian failure as well as reduced breast cancer risk. In males, shorter CAG repeat length is associated with decreased survivorship (mostly in older age) due to higher risk of prostate carcinogenesis, but shorter repeats are associated with increased reproductive fitness (manifested mainly in youth) via increased viability of sperm, reduced risk of Kennedy's disease and perhaps increased reproductive opportunities due to phenotypic traits that are more attractive to females.

### Other putative antagonistically pleiotropic alleles

The examples of antagonistic pleiotropy cited above demonstrate the widespread nature of this phenomenon in terms of physiological systems and tissues as well as mechanisms of action. The previous examples are based on specifically characterized genes; other possible occurrences of antagonistic pleiotropy in humans have been suggested, based upon correlations and less comprehensive data. Further investigation of the proposed cases of antagonistically pleiotropic alleles below seems warranted in order to round out our understanding of the pervasiveness of this phenomenon.

Recent evidence suggests that there may be a link between Triose Phosphate Isomerase (TPI) deficiency, a disease that causes a number of severe symptoms (chronic hemolytic anemia, cardiomyopathy, susceptibility to infection and severe neurological dysfunction), but may also confer oxidative stress resistance. TPI deficiency in yeast confers a protective benefit by reducing oxidative stress during cellular metabolism [56], which suggests that humans who are heterozygous for the mutant TPI allele may also experience this benefit. However, more definitive evidence involving vertebrate models, if not human models, are necessary to further validate this hypothesis.

Tay Sachs disease, a severe neurodegenrative disease due to a number of different mutations described in the HEXA gene which encodes the alpha-subunit of the lysosomal enzyme beta-N-acetylhexosaminidase, has been proposed to owe its surprisingly high frequency to the benefit provided by protection from tuberculosis during the historical process of urbanization [57,58].

Hemochromatosis, a well-known blood disease, has a very clear genetic basis in its manifestation and progression [59] and is often the result of many different potential mutations to different genes with the most common mutation being the C282Y mutation of the HFE gene. Evidence suggests that individuals heterozygous for the C282Y mutation in the HFE gene may experience benefit due to more efficient dietary iron absorption and via increased resistance to typhoid fever and tuberculosis, diseases that became more prevalent with the rise of urbanization [59].

Phenylketonuria (PKU) may also display evidence of selection by antagonistic pleiotropy. Woolf [60] presented data which suggests that heterozygote females may experience a lower chance of mold infection induced miscarriages; potentially an important advantage prior to modern methods of food sanitation and storage. Although the PKU causing allele is likely to be purely deleterious today, its historical heterozygote advantage may account for its current prevalence. The sample of PKU individuals referenced in that study was small and included only individuals in Ireland and Scotland where mold problems have have been more severe than elsewhere, alternative explanations may be necessary to explain the global distribution of PKU.

Another putative example involves hepatocellular carcinoma (HCC), a well-known cancer, and the PTNP11 gene. The wild type allele for PTNP11 is proto-oncogenic and produces the shp2 protein. In its wild-type form, PTNP11 aids in the prevention of HCC but may also contribute to the manifestation of leukemia. Chapeau and colleagues showed that mice and human Leukemia cases are associated with a mutation that leads to the overexpression of the PTNP11 gene [61]. They investigated mouse models with the PTNP11 allele knocked out to simulate under-expression of the gene and the mice were less prone to develop leukemia, but many developed HCC [61]. This data suggests an antagonistic relationship between the levels of PTNP11 expression and the development of leukemia and HCC; further studies must be performed to properly ascertain this same relationship in humans.

Even psychological disorders may be subject to selection of antagonistically pleiotropic alleles. Schizophrenia is believed to have a heritable component with alleles implicated for genetic susceptibility reported to be located in chromosomal regions 6p24-p22 [62] and perhaps 11q21-22[63]. Other studies have shown that although schizophrenic individuals themselves have reduced reproductive success, their unaffected relatives often have higher relative fertility [64], indicating a potential advantage conferred by alleles associated with schizophrenia when those are present as single copies or in different genetic backgrounds (i.e., different epistatic interactions).

Osteoprogesterin (OPG) is a cytokine receptor protein that has a role in malignant tumor progression and modulation of bone density. OPG is encoded by the tumor necrosis factor receptor superfamily member 11B (TNFRSF11B) gene [65]. Overexpression of the TNFRESF11B gene has been associated with several epithelial cancers [66] and another study has suggested that OPG does, in fact, have a modulatory role in tumor angiogenesis [67]. Furthermore, Ito et al [66] have suggest that overexpression is positively correlated with the severity of the associated epithelial cancer (i.e., malignancy increases with the concentration of OPG in the blood). A high level of OPG has also been shown to decrease the likelihood of bone disease; Samuelson and colleagues presented a human clinical study in which a higher than normal level of OPG was associated with stronger bones in both men and women [68]. OPG may act as a promoter of osteoblast activity by reducing the number of activated osteoclasts in the blood. Human clinical studies have shown that there is an association in increased Bone Mineral Density (BMD) in individuals who have higher circulating levels of OPG in their blood plasma. Such costs and benefits associated with the TNFRSF11B gene would be an example of antagonistic pleiotropy, but further evidence, including the identification of the specific genetic polymorphism responsible, is needed to support this hypothesis.

For these less characterized cases of antagonistic pleiotropy, and other cases not identified yet, the genetic mechanism providing the advantage may be straightforward or more complex. The most straightforward mechanism is overdominance (heterozygote advantage), but incomplete penetrance of the deleterious aspect of the alleles or epistatic interactions that differ in different genetic backgrounds may also be important for the full fitness ramifications of disease alleles. The latter two mechanisms are more subtle and difficult to recognize or demonstrate statistically and this may contribute to the unresolved nature of the examples described in this section.

Given the number of diseases caused by antagonistically pleiotropic alleles that have been identified to date and the potential complexity of the mechanisms providing the benefits, many more genetic disorders may exist that have yet to be identified as antagonistically pleiotropic.

## Discussion

We have presented a number of examples where antagonistic pleiotropy has been demonstrated (Table 1) or proposed (Table 2) for disease causing alleles. We feel that antagonistic pleiotropy may provide a potential explanation for the sustained prevalence of many disease-associated alleles in the human population and may be a widespread phenomenon. Because individuals who possess these alleles may have an equal or comparable relative fitness despite their allele's deleterious effects (or the increased risk of experiencing deleterious effects) we predict that pathology-associated alleles will continue to be present in successive generations despite their apparent maladaptive nature.

Although some of the studies cited predict an increase in the frequency of these pleiotropic alleles (e.g.,[15]), whether or not the frequencies of these alleles will increase in successive generations is influenced by changing environmental, medical and social conditions which alter the negative and positive aspects of genetic fitness differences. For example, chelation treatments for Beta-thalassemia have shown great benefit, changing the life expectancy of homozygotes from a few years after birth to as high as 55 years of age [69]. Another example is seen in CF patients where improved medical care in modern times has increased life expectancy of CF individuals from infancy to mid-reproductive age. Even though mortality is still usually experienced before the onset of post-reproductive age, this increase in life-expectancy can have a rather large impact on the reproductive fitness of CF individuals. With the deleterious effects on mortality reduced by modern medicine and the benefits intact these disease alleles would now have a higher net advantage and be expected to rise in frequency more than would have been possible prior to modern medicinal techniques.

On the other hand, since many of the benefits we have discussed involve resistance to diseases that modern medicine will render rarer or more treatable, the benefits may also be reduced and the net change in the fitness provided by these antagonistically pleiotropic alleles is less clear. In fact, because many of the benefits we identified involve resistance to pathogens that are now treated effectively, we may expect these advantageous effects to be reduced and natural selection to be more effective at reducing the frequencies of these alleles in the future. This evolutionary process is typically very slow however, and even if the alleles become purely deleterious, the elimination of these alleles would take a long time.

One readily noticeable feature of the examples of antagonistic pleiotropy we identified is the overabundance of advantageous effects involving malaria; we believe that such bias toward examples involving malaria is due primarily to two major reasons. First, there is a bias in favor of the search for, and recognition of, malaria resistance benefits due to the high profile of Beta-Thalassemia and Sickle-Cell disease which are both such well-known examples of over-dominance that they are often taught in the introductory biology curriculum that medical researchers experience. Such previous exposure pre-disposes epidemiological researchers to consider malaria foremost when investigating explanations for the high frequency of disease alleles. For example, a recent epidemiological study was able to generate a highly detailed map of the distribution of malaria cases worldwide [28]. In accordance with expectations, the highest prevalence of malaria incidences occurred in Mediterranean and African regions, displaying a 58% prevalence rate in 2010. This is an increase since 2002 from a prevalence rate of 56.9%. We, therefore, expect the highest frequencies of malaria resistance mutations to be found in these regions and studies such as this one indicate the level of attention paid to this disorder. Second, the ease with which heterozygous advantage can be demonstrated with these diseases can also be a contributing factor. Malaria is mainly confined to Mediterranean and tropical regions of the world and such a clear geographical distribution allows for a relatively straightforward comparison of allele frequencies in regions likely and unlikely to benefit from malaria protection. Malaria is also a very common and serious disorder overall, leading public health agencies to collect detailed data and provide funding for research related to understanding this disease.

Recognition of the advantageous effects of other disease alleles may be harder to demonstrate experimentally if the advantage provided by the allele does not have a clear and qualitative effect (e.g., if the advantage is only a reduced risk of a disorder rather than a complete elimination of a condition). Such advantageous effects may not be recognized except in studies with large sample sizes in which many aspects of the health of the subjects are recorded. The recent rise of SNP-based association studies may lead to many more discoveries, but only if researchers are measuring a wide range of fitness components. In the ALOX15/BMD example described above [39], the pleiotropy of the identified allele was only recognized because the effects both involved the measured trait. The increased reproduction associated with the Huntington's disease allele was only demonstrable by measuring the number of offspring born to sufferers [15].

For these reasons we recommend that many more variables be measured when conducting studies of associations between alleles and medical conditions that are typically included in published analyses. Life history variables such as total reproduction or age at first reproduction are directly related to fitness and advantageous genetic effects may be made apparent if these values are recorded. We also suggest that additional health relevant statistics such as blood pressure, cholesterol or BMI be measured and included in studies of associations between alleles and medical conditions as they may reveal otherwise overlooked advantageous aspects of allelic differences. Measurement of additional variables carries the risk of false-positives, but follow up studies can be used to minimize that risk whereas failure to record data leads to missed effects that would not be discovered later.

Antagonistic pleiotropy may also have been overlooked because the benefits of these alleles may not be large enough to be readily noticeable when compared to the detrimental effects. A brief mathematical illustration using standard techniques from population genetics (i.e., Hardy-Weinberg style formulations of equilibrium allele frequencies) demonstrates that even minor benefits can result in moderate equilibrium allele frequencies in the case of overdominance (heterozygote advantage). First, define two alleles, «A» and «a», as the wildtype and the mutant allele with relative fitness values for the three genotypes of w(AA) = 1, w(Aa) = 1+S_adv_, w(aa) = 1-S_del_. For example, a value of S_adv _= 0.1 would indicate a 10% higher fitness for heterozygotes whereas a value of S_del _= 0.2 indicates a 20% lower fitness for homozygous for the «a» allele - a value of S_del _= 1 would be an allele that is completely lethal in homozygotes. Using the terms above, and assuming that reproductive decisions are not biased by assortative mating or other factors aside from selection on the alleles, we can derive the following formulae for the equilibrium frequencies of the «a» allele and «aa» genotype:

$$\text{f}\left(\text{a}\right)={\text{S}}_{\text{adv}}∕\left(\text{2}{\text{S}}_{\text{adv}}+{\text{S}}_{\text{del}}\right),\;\text{f}\left(\text{aa}\right)={{\text{S}}_{\text{adv}}}^{2}∕{\left(\text{2}{\text{S}}_{\text{adv}}\text{ + }{\text{S}}_{\text{del}}\right)}^{2}$$

**Figure 1:**
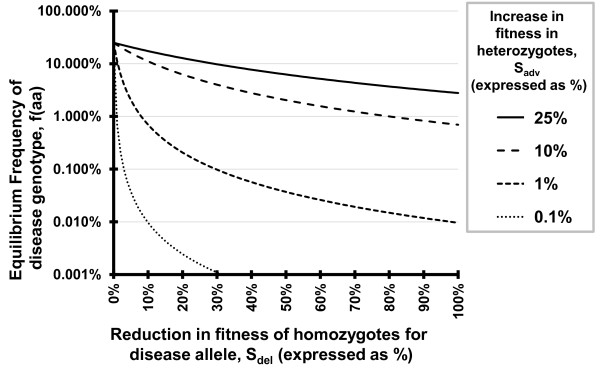
**Expected equilibrium frequency of the disease phenotype caused by an overdominant antagonistically pleiotropic allele**. Equilibrium frequencies for heterozygous advantages, S_adv_, of 0.1%, 1%, 5% and 25% are shown for a range of homozygous deleterious effects, S_del_, ranging from 0% (no deleterious effect) to 100% (allele is lethal or homozygotes are always sterile). Note the Log_10 _scale on the Y-axis.

**Figure 2:**
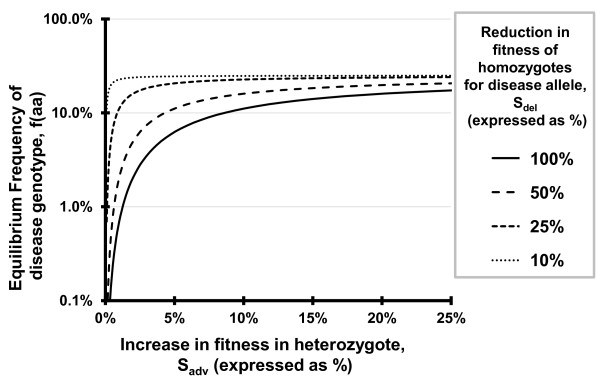
**Expected equilibrium frequency of the disease phenotype caused by an overdominant antagonistically pleiotropic allele**. Equilibrium frequencies for homozygous disadvantages, S_del_, of 100% (completely lethal or always causing sterility), 50%, 25% and 10% are shown for a range of heterozygous advantageous effects ranging from 0% (no advantage) to 25% (a moderate, but potentially unrecognized, advantage). Note the Log_10 _scale on the Y-axis.

Figures 1 and 2 show predicted equilibrium frequencies of the homozygous deleterious genotype for a wide range of realistic parameters. It may seem unlikely that situations in which the advantages plotted in Figures 1 and 2 are not well known, but selective advantages as high as the 25% shown may be unrecognized - the 39% advantage reported for individuals with the Huntington's allele relative to their unaffected siblings [15] was not obvious prior to study [59]. Even in cases in which the heterozygous advantage is minimal (approximately 1%) and the allele is lethal in the homozygous state, natural selection is expected to act to maintain the alleles and result in long-term frequencies of afflicted individuals higher than 1 in 10,000 instead of acting to remove them from the population. This conceptual example illustrates the potential for selection to maintain alleles at medically relevant frequencies even while the benefits conferred may be subtle enough as to be largely unnoticed in a clinical setting.

Selective benefit is not required for the observed presence of deleterious alleles in a population; purely deleterious alleles that confer no advantages persist in populations over time due to recurrent mutations countering the process of elimination by selection. Similar calculations to those above result in predicted equilibrium frequencies much smaller however; ranging from genotypic frequencies of approximately 0.0001% for lethal alleles up to 0.01% for alleles that incur an essentially unnoticeable 1% fitness reduction.

These calculations lead us to predict that from the wide array of known genetic disorders, it is the ones with the highest frequencies that we predict to be most likely to have antagonistically pleiotropic benefits. The process of antagonistic pleiotropy may therefore be highly relevant for understanding the genetic disorders most commonly experienced (i.e., those that tend to result in the highest burden to society). For more complex cases of antagonistic pleiotropy which do not fit the overdominant model examined here, we would generally expect the beneficial aspects of each allele's actions to be comparable to the deleterious effect in order to maintain the allele in the long term - this leads us to predict that we would see antagonistically pleiotropic effects more often for serious medical conditions in which the deleterious effects are more severe.

For these reasons, rather than being a process that is involved in the etiology of a random subset of all genetic disorders, we expect to see antagonistic pleiotropy overrepresented in the diseases that are the most common and have the most severe consequences.

Finally, if antagonistic pleiotropy is indeed as widespread as our reviewed findings and mathematical calculations suggest, the prevalent nature of this phenomenon presents two major implications for the research and treatment of human genetic disease.

First, identification of pleiotropic alleles that confer disease resistance or other benefits and elucidation of their mechanisms of action may aid medical and patho-physiological research. For example, in the case of Tay Sachs, if the hypothesized anti-tuberculosis benefit provided by this allele is demonstrated then identification of the exact cause of this benefit may be useful for the development of novel treatments for tuberculosis - a serious need now that more and more drug resistant strains have evolved. Advances in understanding physiology may also arise from identification of benefits unrelated to disease resistance; a full elucidation of the genetic aspects that contribute to the process by which Huntington's sufferers produce more offspring may prove useful for infertility treatment.

Second, if antagonistically pleiotropic effects are pervasive then this suggests that we use caution when we design genetically-based treatments for diseases. One currently proposed method for treating or preventing cancer is silencing gene expression via interference RNA [70,71]; if such mechanisms completely silence the expression of an antagonistically pleiotropic allele this would lead to a reduction not only in the deleterious effect but also in any beneficial effects. By eliminating a problem we may also eliminate a benefit. For example, silencing an allele that increases the density of AR receptors may reduce cancer risk, but may also cause physiological changes that result in the patient's phenotypic traits becoming less attractive to members of the opposite sex. Especially since the main use of the identification of genetic gene-disease associations in the future will be to decide on prophylactic treatment, a full understanding of the risks and the benefits of undergoing such gene-targeted treatment is essential to ethical patient care. In cases in which a benefit is unknown (but we argue, may well exist), the unknown consequences of turning off potentially pleiotropic alleles implies that a different overall approach should be considered to avoid potential unwanted or disastrous side-effects. Rather than just focusing on a culprit disease allele and silencing it, selectively inhibiting the allele's deleterious pathway while allowing the beneficial pathway to persist becomes a more responsible, albeit more difficult, course of action.

## Conclusion

We have identified a number of empirical examples where antagonistic pleiotropy has been demonstrated and more where such pleiotropy is likely. A population genetic model suggests that alleles with extremely minor fitness advantages may maintain otherwise deleterious alleles at medically relevant frequencies. Based on the availability of this information despite no concerted research effort to gather it and the mathematical argument we feel that such antagonistic pleiotropy may be a widespread phenomenon. If such pleiotropy is widespread this opens avenues for the identification of novel physiological pathways by the study of the pleiotropic effects of disease alleles and suggests that caution be taken when designing genetic therapies to eliminate the effects of genes lest their beneficial effects be removed as well.

## Competing interests

The authors declare that they have no competing interests.

## Authors' contributions

AC conceived the initial project and provided the population genetic argument. AN obtained the data used and wrote the first draft of the manuscript. Both authors participated in writing of the final manuscript, addressing all revisions and approved the final manuscript.

## Pre-publication history

The pre-publication history for this paper can be accessed here:

http://www.biomedcentral.com/1471-2350/12/160/prepub
